# Clonal evolution is a prognostic factor for the clinical progression of monoclonal B-cell lymphocytosis

**DOI:** 10.1038/bcj.2017.77

**Published:** 2017-08-25

**Authors:** I V Kostopoulos, G Paterakis, D Pavlidis, E Kastritis, E Terpos, O E Tsitsilonis, S I Papadhimitriou

**Affiliations:** 1 Hematology Laboratory, ‘G. Gennimatas’ Athens General Hospital, Athens, Greece; 2 Section of Animal and Human Physiology, Department of Biology, National and Kapodistrian University of Athens, Athens, Greece; 3 Flow Cytometry Laboratory, ‘G. Gennimatas’ Athens General Hospital, Athens, Greece; 4 Department of Clinical Therapeutics, School of Medicine, National and Kapodistrian University of Athens, Athens, Greece

Monoclonal B-cell lymphocytosis (MBL) has attracted intensive research as the prelude of chronic lymphoproliferative disorders, mainly chronic lymphocytic leukemia (CLL).^1^ According to current criteria, MBL is a preclinical condition characterized by monoclonal B-cell expansions at small concentrations (<5 × 10^9^ cells/l) in the peripheral blood of otherwise healthy individuals.^2, 3^ It is now obvious that MBL is a highly heterogeneous entity regarding the immunophenotypic characteristics and the B-cell clone burden.^4, 5, 6^ In immunophenotypic terms, MBL is distinguished into three main categories: (i) CLL-like (CD5^+^CD20^dim^CD23^+^sIg^low^) accounting for 70–75% of all cases; (ii) atypical (CD5^+^CD20^bright/+^), mainly CD23^dim/^^−^; and (iii) CD5^neg^ MBL.^2, 3^ Based on the number of monoclonal B-cells, MBL is divided into low and high-count, each with a clearly different clinical course.^5, 6, 7^ Low-count MBL is a non-progressive entity with a normal absolute B-cell count, whereas high-count or ‘clinical’ MBL (cMBL) is characterized by absolute lymphocytosis and progresses to CLL at a rate of ~1–2% per year.^8, 9^

The prevalence of CLL-like MBL is 10–100-fold higher than that of CLL, indicating that most cases—even within the high-count category—do not evolve to overt disease.^8, 9, 10, 11^ However, the biological features and molecular events which may contribute to the transition into the clinical state are far from being completely elucidated. Particularly, the available information on the clinical progression of non CLL-like MBL is scanty.^12, 13^ To improve our understanding of these processes we performed a prospective study on a large series of 227 cMBL cases of all three phenotypic categories testing for possible changes occurring during the natural course of MBL. These changes together with all available clinical parameters were further evaluated for their potential role in lymphomagenesis during a long-term follow-up (median 76 months).

All individuals included, attended the outpatient clinics of our hospital between January 2001 and January 2015 and characterized as MBL of any type according to current diagnostic criteria. They had no history or evidence of a hematological/solid neoplasia or autoimmunity and provided written informed consent to use laboratory data for research studies. The study followed the rules of the Interval Review Board and adhered to the declaration of Helsinki.

To avoid a mixed cohort with low-count MBL, we excluded cases with fewer than 0.5 × 10^9^ clonal B cells/L. All subjects underwent a regular hematologic follow-up every 6–12 months (median number of follow-up visits: 5; range 2–16). The MBL phenotype was characterized at diagnosis by flow-cytometry and the same cytometric assessment was repeated in cases with evidence of disease progression. A detailed cytogenetic evaluation was performed by fluorescent *in situ* hybridization at presentation and repeated at least once for all individuals still at the MBL stage, no sooner than 18 months after initial testing (median interval to repetition: 26 months; range 18–48). Disease progression was based on B-cell cut-off limit of >5 × 10^9^ cells/l (persisting for 3 months), and/or an increase in bone marrow infiltration rate >20%, and/or the appearance of lymphadenopathy/organomegaly, detected either clinically or on ultrasound/computed tomography scans. Differences in time-to-event analysis were evaluated by log-rank statistics and multivariate analysis was performed by Cox proportional hazard regression. In case of continuous variables, the optimal cutoff points predicting the progression to overt disease were defined using the receiver–operator characteristic curves.

The flow-cytometry analysis showed a CLL-like phenotype in 130 subjects (57.3%), an atypical one in 42 (18.5%) and a CD5^neg^ one in 55 (24.2%). The three groups showed differences in some biological and hematological features such as the increased expression levels of ZAP70 in the non-CLL-like groups, and the significant lower presence of B-cells in the CD5^neg^ group both in absolute counts and qualitatively when measured as a percentage (%) of the total lymphocytes (Table 1). Our broad fluorescent *in situ* hybridization analysis revealed abnormalities in 124/227 cMBL cases (54.6%) at the initial examination with del(13q14) being the most frequent (72/227, 31.7%). Overall, CD5^neg^ MBL exhibited less often cytogenetic aberrations than the other groups (36.4% in CD5^neg^ vs 63.1% in CLL-like and 52.4% in atypical MBL, *P*=0.038) and most importantly, each category showed a clearly distinct cytogenetic pattern. Particularly, biallelic del(13q14) as a sole abnormality and concurrent monoallelic/biallelic del(13q14) were found only in CLL-like cases, t(11;14) detected only in atypical and del(7q31) only in CD5^neg^ clones. In contrast, trisomy 12 was the only common finding among the 3 groups, whereas no del(6q23) or t(18q21) were detected in any of the analyzed samples.

In general, the usual cytogenetic aberrations detected in CLL or other chronic lymphoproliferations are regarded as independent genetic events, so the presence of multiple aberrations may be considered as signs of clonal evolution. The same holds also true for homozygous 13q deletions.^14^ Accordingly, 19 cMBL cases, mostly of the CLL-like group, showed evidence of clonal evolution (13 with homozygous or concomitant mono/biallelic del(13q14) and six cases with multiple abnormalities). However, the genomic instability was further highlighted by the cytogenetic re-evaluation performed in each participant, while still at the cMBL phase. Particularly, the cytogenetic re-evaluation revealed 14 cases (11 CLL-like, two atypical and one CD5^neg^) showing novel abnormalities which were absent at presentation. Most of these cases acquired del(13q14) or +12 on a previous normal background (six and three cases respectively); in one case the hemizygous del(13q14) evolved to concomitant hemizygous/homozygous loss, in one cMBL the initial concomitant mono/biallelic del(13q14) evolved to homozygous loss and in another case +12 appeared in addition to del(13q14) already detected at diagnosis. The thirteenth case included the novel appearance of del(13q14) in the pre-existing +12 and the last case acquired a gain in 18q21 locus in addition to del(13q14) found initially.

The prospective nature of this study and our long-term monitoring allowed us to evaluate parameters which have an important role in the clinical progression of MBL. To date, 78 cMBL cases (34.3%) have evolved to overt disease (46/130 CLL-like, 15/42 atypical and 17/55 CD5^neg^ cases) and 27 of them (11.9%) have required treatment. Of the three groups CD5^neg^ cMBL had the most favorable profile (median time-to-progression: 84 months vs 66 months in CLL-like and 45 months in atypical, Figure 1a), which could be explained by the lower prevalence of genetic lesions compared to the other MBL subsets. Furthermore, the increased T/NK component in the CD5^neg^ cases indicate an active role of microenvironmental ‘bystander’ immune cells that dynamically interact with the CD5^neg^ cells and may restrain the clone in an indolent state.^15^

With regard to subgroup analysis, we searched for correlations between time-to-progression and clinical or biological features at diagnosis within each phenotypic subset. The variables tested were age, sex, WBC, ALC, B-cell count, T-cell count, platelet count, hemoglobin level, bone marrow infiltration rate, CD38 ⩾30%, ZAP70 ⩾20%, initial fluorescent *in situ* hybridization findings, immunoglobulin heavy chain variable region gene mutational status and the presence of clonal evolution. The higher B-cell count correlated with shorter time to disease progression in all phenotypic subsets, implying that common risk factors may operate in all different forms of the preclinical cMBL status. With the B-cell count treated as a continuous variable, we tried to define the optimal cutoff risk points in each category. A running log-rank test with a step-increase of 100 cells/μL revealed two peaks in CLL-like MBL; cases with an initial B-cell count of more than 3900/μl carried a high risk of progression (median time: 37 months), those with a count of 2000–3900/μl had an intermediate risk (median time: 58 months), whereas those with a count below 2000/μl showed a significantly lower probability (median: 116 months) (*P*<0.001 for all comparisons). Following the same approach, we found one peak of 3600/μl for atypical (*P*=0.007) and one peak of 3200/μl for CD5^neg^ MBL cases (*P*=0.005) (Figures 1b–d).

Of the remaining variables, only clonal evolution was independently associated with a high risk of progression to clinical disease (hazards ratio: 2.25, 95% CI:1.16-4.38 *P*=0.017). Since clonal evolution was mainly detected in the CLL-like cases, risk analysis was restricted to this subset for better homogeneity, although statistical significance was not affected when all cases were included. In particular, individuals with clonal evolution had a shorter time-to-progression (median: 44 months), when compared to those who were cytogenetically stable (median: 82 months, *P*<0.001, Figure 1e). Interestingly, the presence of clonal evolution could clearly discriminate between two subgroups with significantly different risks of progression among patients at the intermediate-risk category according to B-cell count (2000–3900 B-cells/μl) (median time: 47 vs 77 months, *P*<0.001) (Figure 1f). Finally, the negative impact of this parameter was also identified in time-to-treatment analysis, since cases with clonal evolution at the preclinical stage, showed both an increased rate for treatment requirement in our monitoring period and a significant shorter time-to-treatment when compared with the rest of the cohort (Figure 1g).

In summary, the three phenotypically defined MBL groups have distinct characteristics, but seem to share common features involved in clinical progression. Certain chromosome aberrations occur early in the transformation process but do not seem to affect the risk of progression to the clinical state. On the other hand, the apparent genomic instability, manifested by the acquisition of additional abnormalities, together with increased initial B-cell counts are the strongest determinants of disease progression. The combination of these two variables could better stratify MBL subjects into more precise prognostic subgroups.

## Figures and Tables

**Figure 1:**
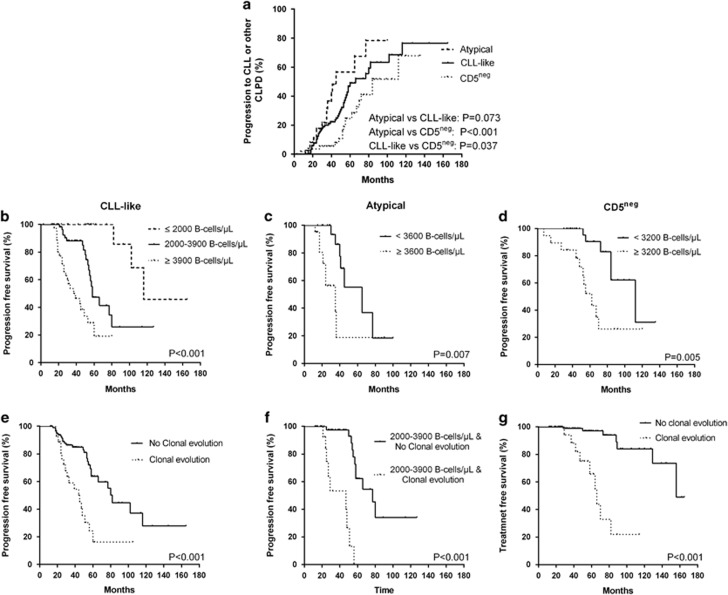
Risk of progression from MBL to CLL or other CLPDs. (**a**) Time-to-progression according to the phenotypic category. (**b**–**d**) Progression-free survival according to absolute B-cell count for CLL-like (**b**), atypical (**c**) and CD5^neg^ (**d**) MBL. (**e**) Progression-free survival according to the presence of clonal evolution. (**f**) Progression-free survival for CLL-like MBL subjects with 2000–3900 B-cell count at initial diagnosis according to the presence or absence of clonal evolution. (**g**) Treatment-free survival for progressed cases according to the presence of clonal evolution at the preclinical MBL stage. CLPD, chronic lymphoproliferative disorder.

**Table 1:** Clinical, biological and hematological features of individuals enrolled

*Characteristic*	*CLL-like MBL (*N=*130)*	*Atypical MBL (*N=*42)*	*CD5*^*neg*^ *MBL (*N=*55)*	P
Age (years)	64 (26-91)^a^	70 (42–84)	67 (27–92)	0.08
Male sex	65/130 (50%)	28/42 (66.7%)	27/55 (49.1%)	0.139
HB (g/dl)	14.0 (10–17.1)	13.3 (10.4–17.1)	13.3 (10.3–16.9)	0.042^b^
Platelets (× 10^9^/l)	216 (127–353)	221 (113–376)	220 (80–335)	0.801
BM infiltration (%)	11 (0–20)	12 (0-18)	11 (0–20)	0.699
WBC (per μl)	10900 (4150–18000)	10700 (3430–18000)	11300 (4110–31 500)	0.518
ALC (per μl)	6110 (1876–12600)	6069 (2250–9580)	6036 (1060–12 200)	0.508
B-cell count (per μl)	3309 (568–4954)	3373 (718–4948)	2541 (507–4940)	<0.001^c^
B-cell compartment to total lymphocytes (%)	53.8 (13.5–93.5)	59.2 (18.3–76.6)	45.3 (13.8–81.8)	<0.001^c^
B-cells with MBL phenotype (%)	94.5 (45.6–100)	95.2 (73.6–100)	95.8 (72–100)	0.458
FISH abnormality	82/130	22/42 (52.4%)	20/55 (36.4%)	0.004^b^
Normal	48/130 (36.9%)	20/42 (47.6%)	35/55 (63.6%)	
del(13q14)x1	45/130 (34.6%)	9/42 (21.4%)	0/55	
del(13q14)x2	10/130 (7.7%)	0/42	0/55	
del(13q14)x1/del(13q14)x2	3/130 (2.3%)	0/42	0/55	
+12	15/130 (11.5%)	5/42 (11.9%)	6/55 (10.9%)	
del(11q23)	3/130 (2.3%)	0/42	1/55 (1.8%)	
del(17p13)	1/130 (0.7%)	0/42	0/55	
t(11;14)(q13;q32)	0/130	5/42 (11.9%)	0/55	
del(6q23)	0/130	0/42	0/55	
t(14q32)	2/130 (1.5%)	0/42	4/55 (7.3%)	
+3	0/130	2/42 (4.8%)	4/55 (7.3%)	
del(7q31)	0/130	0/42	3/55 (5.5%)	
t(18q21)	0/130	0/42	0/55	
Multiple abnormalities				
del(13q14)x1/del(11q22)	2/130 (1.5%)			
del(13q14)x1/+12	1/130 (0.7%)			
del(13q14)x2/+12/3x14q32		1/42 (2.4%)		
del(13q14)x1/del(17p13)			1/55 (1.8%)	
+12/3x18q21			1/55 (1.8%)	
Mutated IGHV	63/78 (80.8%)	10/12 (83.3%)	12/15 (80%)	0.973
CD38>30%	24/130 (18.5%)	8/40 (20%)	7/52 (13.5%)	0.657
ZAP70>20%	36/120 (30%)	19/35 (54.3%)	25/49 (51%)	0.005^d^

Abbreviations: ALC, absolute lymphocyte count; BM, bone marrow; CLL, chronic lymphocytic leukemia; FISH, fluorescent *in situ* hybridization; Hb, hemoglobin; MBL, monoclonal B-cell lymphocytosis; WBCs, white blood cells.

a Median value (with range in parenthesis) for continuous variables.

b Significance *P*<0.05 between CD5^neg^ and CLL-like MBL.

c Significance *P*<0.05 between CD5^neg^ and CLL-like & between CD5^neg^ and atypical MBL.

d Significance *P*<0.05 between CLL-like and atypical & between CLL-like and CD5^neg^ MBL.
